# Supplementary material for: Parallel Synapses with Transmission Nonlinearities Enhance Neuronal Classification Capacity
Source: bioRxiv. 2024 Jul 4:2024.07.01.601490. Preprint. [Version 1] doi: 10.1101/2024.07.01.601490 (PMC11244940; doi:10.1101/2024.07.01.601490)
Supplement: Supplement 1 [file NIHPP2024.07.01.601490v1-supplement-1.pdf]

bioRxiv preprint doi: https://doi.org/10.1101/2024.07.01.601490; this version posted July 4, 2024. The copyright holder for this preprint (which was not certified by peer review) is the author/funder, who has granted bioRxiv a license to display the preprint in perpetuity. It is made available under aCC-BY 4.0 International license.

Supplementary Materials

Comparison of two types of neural networks for MNIST task

Here we enumerate the total number of parameters in networks with nonlinear parallel synapses and networks with standard linear synapses. We use D_{in} as the number of neurons in the input layer, which is $28 * 28$ for both types of network. D_{out} denotes the number of neurons in the output layer, which is 10 for both types of network. D_{hidden} is the number of neurons in the hidden layer. We choose D_{hidden} as $5, 10, 20$ and 30 for networks with parallel synapses. We set the number of parallel synapses per connection to 3, i.e., $M = 3$. For networks with nonlinear parallel synapse in the hidden-output connection, the total number of parameters is $(D_{\text{in}} + 1)D_{\text{hidden}} + (3MD_{\text{hidden}} + 1)D_{\text{out}}$, including the bias terms. For networks with only single linear synapses, the total number of parameters is $(D_{\text{in}} + 1)D_{\text{hidden}} + (D_{\text{hidden}} + 1)D_{\text{out}}$, also including the bias terms. Therefore, we opt for a slightly larger D_{hidden} for networks with linear synapses to achieve a fair comparison. The parameter counts for the two types of networks are shown below in Table 1.

**Network type**	**#parameters**	**Network type**	**#parameters**
$D_{\text{hidden}} = 5,\ 3$ parallel synapses	4370	$D_{\text{hidden}} = 6$, linear synapses	4780
$D_{\text{hidden}} = 10,\ 3$ parallel synapses	8760	$D_{\text{hidden}} = 11$, linear synapses	8755
$D_{\text{hidden}} = 20,\ 3$ parallel synapses	17510	$D_{\text{hidden}} = 22$, linear synapses	17500
$D_{\text{hidden}} = 30,\ 3$ parallel synapses	26260	$D_{\text{hidden}} = 33$, linear synapses	26245

Table 1: Comparison of parameter numbers in networks with parallel synapses (left two columns) and networks with single linear synapses (right two columns).

Below we record the accuracy of both types of networks on the testing set after 50 epochs of training in Table 2. The standard deviation is calculated from 20 trained networks with different initialization seeds.

**Network type**	**Accuracy**	**Network type**	**Accuracy**	**Gain**
$D_{\text{hidden}} = 5,\ 3$ parallel synapses	$91.42\% \pm 0.06\%$	$D_{\text{hidden}} = 6$, linear synapses	$89.93\% \pm 0.11\%$	**1.49%**
$D_{\text{hidden}} = 10,\ 3$ parallel synapses	$94.92\% \pm 0.06\%$	$D_{\text{hidden}} = 11$, linear synapses	$92.93\% \pm 0.04\%$	**2.00%**
$D_{\text{hidden}} = 20,\ 3$ parallel synapses	$96.41\% \pm 0.05\%$	$D_{\text{hidden}} = 22$, linear synapses	$94.90\% \pm 0.03\%$	**1.51%**
$D_{\text{hidden}} = 30,\ 3$ parallel synapses	$96.77\% \pm 0.03\%$	$D_{\text{hidden}} = 33$, linear synapses	$96.02\% \pm 0.02\%$	**0.75%**

Table 2: Comparison of the classification accuracy in networks with parallel synapses (left two columns) and networks with single linear synapses (middle two columns). The accuracy improvements from using parallel synapses are shown in the right column.

bioRxiv preprint doi: https://doi.org/10.1101/2024.07.01.601490; this version posted July 4, 2024. The copyright holder for this preprint (which was not certified by peer review) is the author/funder, who has granted bioRxiv a license to display the preprint in perpetuity. It is made available under aCC-BY 4.0 International license.

Learned aggregate synaptic function in neural networks

For the network depicted in Fig. 4a (with $D_{\text{hidden}} = 10$, 3 parallel synapses), we can also visualize the activation distributions of individual hidden units, as shown in Fig. 6. The input patterns are from the testing set of the MNIST dataset. For each hidden unit, we collect its activations (before the nonlinear synaptic transmission function) across various input patterns. These activations serve as the input values to the parallel synapses. Each hidden unit is connected to each output unit via a set of parallel synapses. A set of parallel synapses can be represented by their aggregate synaptic function. Thus, for each hidden unit, we can plot all the aggregate synaptic functions connecting this hidden unit to all the output units, which is also visualized in Fig. 6. In addition, Fig. 7 shows the distributions of parameters (slope, amplitude and threshold) in the parallel synapses from the same network.

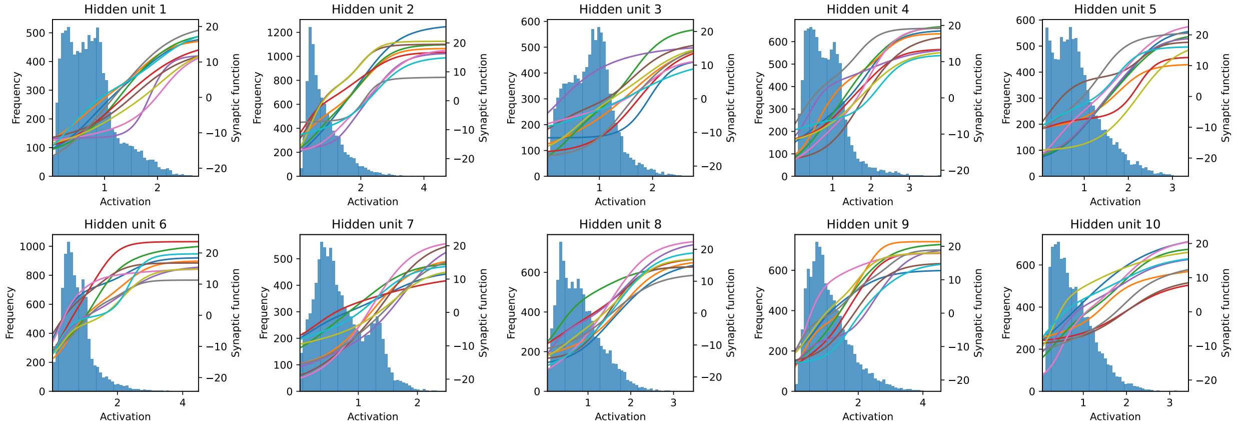

Figure 6: Histograms of hidden unit activations in the two-layer neural network with parallel synapses, overlaid with learned aggregate synaptic transmission functions. The x-axis is the total activation of the corresponding hidden unit. The left y-axis is the frequency of activation values. The right y-axis is the value of the aggregate synaptic function. Each panel corresponds to one hidden unit. The histograms collect activation values from all patterns in the testing set of the MNIST dataset. The lines are aggregate synaptic functions connecting each hidden unit to all output units, with 10 output units in total.

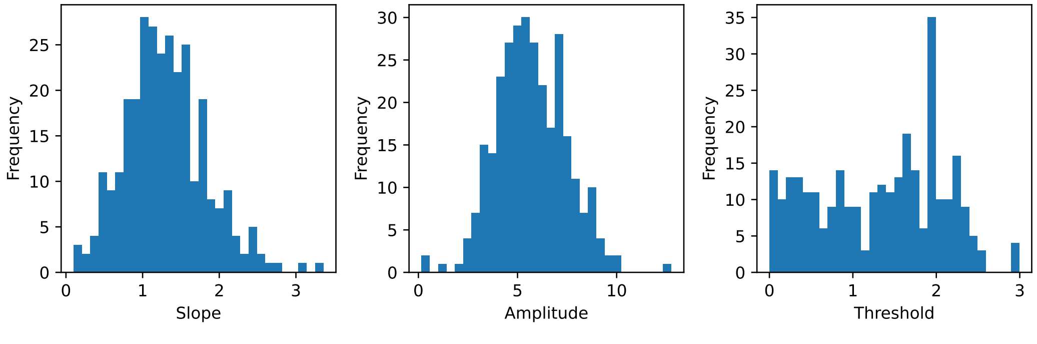

Figure 7: Histograms of parameters in parallel synapses from the same network as in Fig. 4, with slope (left), amplitude (middle) and threshold (right).

bioRxiv preprint doi: https://doi.org/10.1101/2024.07.01.601490; this version posted July 4, 2024. The copyright holder for this preprint (which was not certified by peer review) is the author/funder, who has granted bioRxiv a license to display the preprint in perpetuity. It is made available under aCC-BY 4.0 International license.

**Parameter type**	**Parameter value**
optimizer	Adam [39] with default PyTorch setting
batch size	256
learning rate for parallel synapses	0.02
learning rate for bias in batch normalization	0.01
learning rate for other parameters	0.001
learning rate scheduler	StepLR in PyTorch
step size of learning rate scheduler	5
decrease factor of learning rate scheduler	0.5
number of epochs	50
activation function of hidden layer	Softplus
weight initialization for linear synapses	Kaiming uniform distribution [40]
initialization for amplitude parameters	uniform distribution $\mathcal{U}(2,3)$
initialization for slope parameters	uniform distribution $\mathcal{U}(1,2)$
initialization for threshold parameters	uniform distributino $\mathcal{U}(0,3)$

Table 3: Hyperparameter settings for training neural networks.
